# Development of Janus Cellulose Acetate Fiber (CA) Membranes for Highly Efficient Oil–Water Separation

**DOI:** 10.3390/ma14205916

**Published:** 2021-10-09

**Authors:** Xiaotian Yu, Xian Zhang, Yajie Xing, Hongjing Zhang, Wuwei Jiang, Ke Zhou, Yongqiang Li

**Affiliations:** 1 College of Textiles Science and Engineering (International Silk Institute), Key Laboratory of Advanced Textile Materials and Manufacturing Technology, Ministry of Education, Zhejiang Sci-Tech University, Hangzhou 310018, China; a1521750962@163.com; 2 Engineering Research Center for Eco-Dyeing and Finishing of Textiles, Ministry of Education, Zhejiang Sci-Tech University, Hangzhou 310018, China; z871279@163.com (X.Z.); jessixing118@163.com (Y.X.); hongjingzhang431@gmail.com (H.Z.); jiangww5034@gmail.com (W.J.); zhoukk4399@163.com (K.Z.)

**Keywords:** asymmetric wettability, superhydrophobic, hydrophilic, Janus–CA fiber membrane, centrifugal spinning, oil–water separation

## Abstract

A new type of Janus cellulose acetate (CA) fiber membrane was used to separate oil–water emulsions, which was prepared with plasma gas phase grafting by polymerizing octamethylcyclotetrasiloxane (D4) onto a CA fiber membrane prepared by centrifugal spinning. The Janus–CA fiber membrane was described in terms of chemical structure using Fourier transform infrared spectroscopy (FTIR), X-ray photoelectron spectroscopy (XPS) analysis, energy dispersive X-ray spectroscopy (EDX) analysis and morphology by field emission scanning electron microscopy (FESEM). In this contribution, we examine the influence of spinning solution concentration, spinning speed and nozzle aperture on the centrifugal spinning process and the fiber morphology. Superhydrophobic/hydrophilic Janus–CA fiber membrane was used to separate water and 1,2-dibromoethane mixture and Toluene-in-water emulsion. Unidirectional water transfer Janus–CA fiber membrane was used to separate n-hexane and water mixture. The separation for the first-time interception rate was about 98.81%, 98.76% and 98.73%, respectively. Experimental results revealed that the Janus cellulose acetate (CA) fiber membrane gave a permeate flux of about 43.32, 331.72 and 275.27 L/(m^2^·h), respectively. The novel Janus–CA fiber membrane can potentially be used for sustainable W/O emulsion separation. We believe that this is a facile strategy for construction of filtration materials for practical oil–water separation.

## 1. Introduction

Water and oil are important resources for people’s production and development. However, in recent years, oil development has led to frequent offshore oil spills [1,2]. The traditional oil–water separation method requires a large area of equipment, high energy consumption and low-separation efficiency [3]. The membrane separation method has the advantages of high separation rate, low energy consumption and simple operation [4,5]. Janus membrane, as a derivative of separation membrane, has a great application prospect in the fields of fog collection [6], swappable ion transport [7,8] directional fluid control [9] and membrane separation technology [10,11,12].

Zhu et al. [13] prepared a polyvinylidene fluoride (PVDF) nanofiber membrane via the electrostatic spinning method. By spraying the nanofiber membrane with different concentrations of hydrophobic or hydrophilic SiO_2_ spray at the nanoscale, the highly permeable Janus nanofiber membrane was prepared. Hou et al. [14] designed an anisotropic Janus membrane with an interpenetrating structure in a microscopic state. Common Janus membranes are mainly hydrophobic/hydrophilic Janus membranes and positive/negative Janus membranes. Hydrophobic/hydrophilic membranes are hydrophobic on one side and hydrophilic on the other side. When water droplets are dropped on the hydrophobic side of the membrane, the hydrophobic effect of the membrane surface and the surface tension of the water itself will make the water droplets appear spherical, and the spherical water droplets on the membrane surface was squeezed into the membrane hole. The hydrophilic face has an affinity for water, and it will pull the water droplets to the other side, such that the water is transmitted spontaneously. However, the reverse is irreversible [15]. When water droplets drip from the hydrophilic surface, they will wet the surface of the fiber membrane. The hydrophobic surface has no pull on the water droplets, and the water droplets cannot penetrate to the hydrophobic surface; thus, it has the characteristics of a “fluid diode” [16,17]. We use the hydrophilic material acetate fiber as the substrate, such that only one-sided hydrophobic modification can make the fiber membrane achieve an asymmetric wetting structure. The performance of such fiber membranes for oil–water separation is better than that of homogeneous hydrophobic membranes. The separation of water in the mixed solution can reduce the contamination of the fiber membranes and increase the membrane flux. Wang et al. [18] introduced a new double-function Janus cotton fabric which is superhydrophobic on one side and has polyamine on the other, and they used it to separate oil from the oil–water emulsion.

The above studies have carried out a series of explorations for the preparation and application of the Janus membrane, but the preparation reaction conditions are not easy to control, and the operation is complex, which has a certain influence on the development of the Janus membrane.

Cellulose acetate membranes generally exhibit high hydrophilicity, high water permeability and low membrane fouling tendency [19]. Cellulose is easily available in nature and is the most abundant organic raw material in the world [20], with an annual output of close to 1000 tons [21,22]. Cellulose macromolecules have special properties such as regular structure, large molecular weight, and active hydroxyl groups, and different reactions can be carried out on them. It is also easily obtained from renewable resources [23,24,25]. These properties help to use cellulose as a precursor for chemical modification. Currently, hydrophilic membrane materials such as cellulose acetate have excellent anti-fouling properties and have been widely used in oil/water membrane separation [26,27,28]. In the past ten years, various methods have been developed to design super-wetting membranes for efficient oil and water separation [29], such as electrospinning, physical mixing, surface graft polymerization, surface coating, chemical oxidation and plasma treatment [30,31].

Although cellulose acetate performed well in oil–water separation, Shoba et al. [32] found that the membrane flux was low when the cellulose acetate concentration reached the optimal value of 17.5 wt%; thus, we looked for a new spinning method to prepare cellulose acetate membrane. Centrifugal fiber spinning can be considered as a promising alternative to traditional spinning technologies such as electrospinning and meltblown spinning. In electrospinning, the polymer fluid jet is pushed from the nozzle and exposed to a high electric field. Meanwhile, the charged jet will be whipping and bending instabilities, resulting in thinning of the jet and evaporation of the solvent. In addition, due to the relatively low production rate [33,34] and the demand for high voltages, it is necessary to find a better alternative spinning technology. This is a simple technology based on centrifugal force to provide higher productivity at a lower cost. This leads to a technology that is environmentally and economically superior to other technologies. Plasma technology has the characteristics of energy saving, environmental protection, and it is fast and efficient [35]. While modifying the fiber surface, it will not damage the excellent quality and mechanical properties of the material itself, and the treatment only involves 30–50 nm on the surface of the material. Recently, Hussain et al. [36] realized the preparation of cellulose acetate membrane with an oil–water separation rate of 70%. Inspired by the Janus membrane, we successfully improved the oil–water separation rate of the membrane greatly by modifying it by plasma grafting on one side. In addition, the membrane flux was improved.

In this article, the CA fiber membrane was efficiently prepared by the centrifugal spinning method. The Janus–CA fiber membrane was prepared by plasma grafting D4. This research aims to further improve the performance of a cellulose membrane for oil–water separation. Meanwhile, the purpose of this research is the synthesis, characterization and application of a Janus cellulose acetate membrane in the treatment of oily wastewater. The results of this study have certain reference significance for the controllable preparation of Janus fiber membranes and the separation of oily wastewater.

## 2. Materials and Methods

### 2.1. Materials

CA with a molecular weight of 100,000 g/mol was supplied from Acros Organics (Fisher Scientific Worldwide (Shanghai) Co. Ltd., Shanghai, China). Toluene (analytically pure, Sinopharm Chemical Reagent Co. Ltd., (Shanghai, China). D4 (90%, CAS#556-67-2) was purchased from Zhejiang Quzhou Jun Shun Silicone Co. Ltd., (Quzhou, China). Acetone (analytically pure, Zhejiang Sanying Chemical Reagent Co. Ltd., Hangzhou, China). N-hexane (analytically pure, Hangzhou Gaojing Fine Chemical Co. Ltd., Hangzhou, China). 1, 2-dibromoethane, methylene blue, Disperse Red FB and Span 80 were of analytical grade and purchased from Shanghai McLean biochemical technology Co. Ltd., (Hangzhou, China). Oxygen (99.9%) was supplied by Hangzhou Electrochemical Group Co. Ltd., (Hangzhou, China).

**Figure 1:**
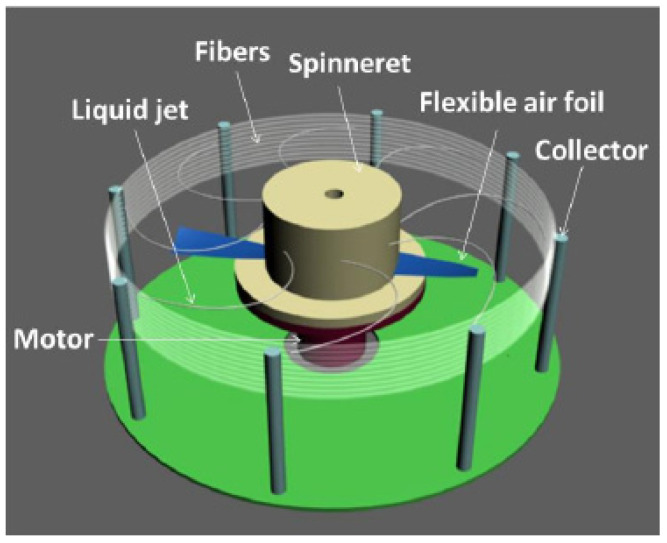
Structure drawing of a centrifugal spinning machine.

The low-temperature plasma was produced by a conventional radio frequency (RF) capacitance coupling plasma device (Model: HD-1A, Changzhou Changtai Plasma Technology Development Ltd., Changzhou, China). The centrifugal spinning equipment is shown in Figure 1. The spinning head is fixed on the motor shaft by screws, the spinneret is located on the sidewall of the spinning head, and the receiving rod is used to receive the fibers.

### 2.2. Preparation of Superhydrophobic/Hydrophilic Janus–CA Fiber Membrane

The CA fiber membrane was prepared by the centrifugal spinning method, and then the single-sided plasma gas phase grafting of the CA fiber membrane with D4 monomer was used to prepare the superhydrophobic/hydrophilic and the unidirectional water transfer Janus–CA fiber membrane. The specific preparation method is as follows:

(1) Preparation of centrifugal spinning CA fiber membrane. CA with a concentration of 16–20 wt% was added into a mixed solvent with a mass ratio of acetone and dimethyl sulfoxide (DMSO) of 7:3. Then, it was stirred at 55 °C on a magnetic stirrer for 12 h (500 r/min) and started to centrifugal spinning. The diameter of the spinning head was 0.3–0.5 mm, and the spinning speed was 6000, 8000, and 10,000 r/min. The spinning parameters were explored to realize the controllable preparation of the centrifugal spinning of CA fiber membrane.

(2) Preparation of Janus–CA fiber membrane. The CA fiber film was placed on the protective layer to protect one side and placed in the plasma machine. The D4 gas was passed when evacuating to 10 Pa, and the pressure of D4 gas was set to 15 Pa for 3–10 min. The power during the whole process was 40–100 W to perform single-sided hydrophobic finishing on the spun PVDF fiber membrane. The super-hydrophobic/hydrophilic and unidirectional liquid transport Janus–CA fiber membranes were prepared by controlling the plasma treatment parameters.

### 2.3. Membrane Characterizations

To investigate the morphology of the membrane, surface morphology of the membrane was then studied using field emission scanning electron microscope (Ultra 55, Zeiss, Germany). The chemical structure of the obtained Janus–CA fibers was assayed with a Fourier transform infrared (FTIR) spectrometer (Nicolet 5700 type, Thermo Fisher Scientifc, Waltham, MA, USA). X-ray photoelectron spectroscopy (K-alpha, Thermo Fisher Scientific, Waltham, MA, USA) was used to analyze the surface elements of the fiber membrane.

### 2.4. Water Contact Angle Test

A video contact angle tester (DSA20, Krüss, Germany) was used to test the surface water contact angle of the fiber membrane before and after the plasma treatment. A volume of 3 μL of deionized water was designed to be dropped on the surface of the fiber membrane. Five points were randomly selected for each sample for testing, and the average value of the test data was calculated.

### 2.5. Membrane Flux Test

**Figure 2:**
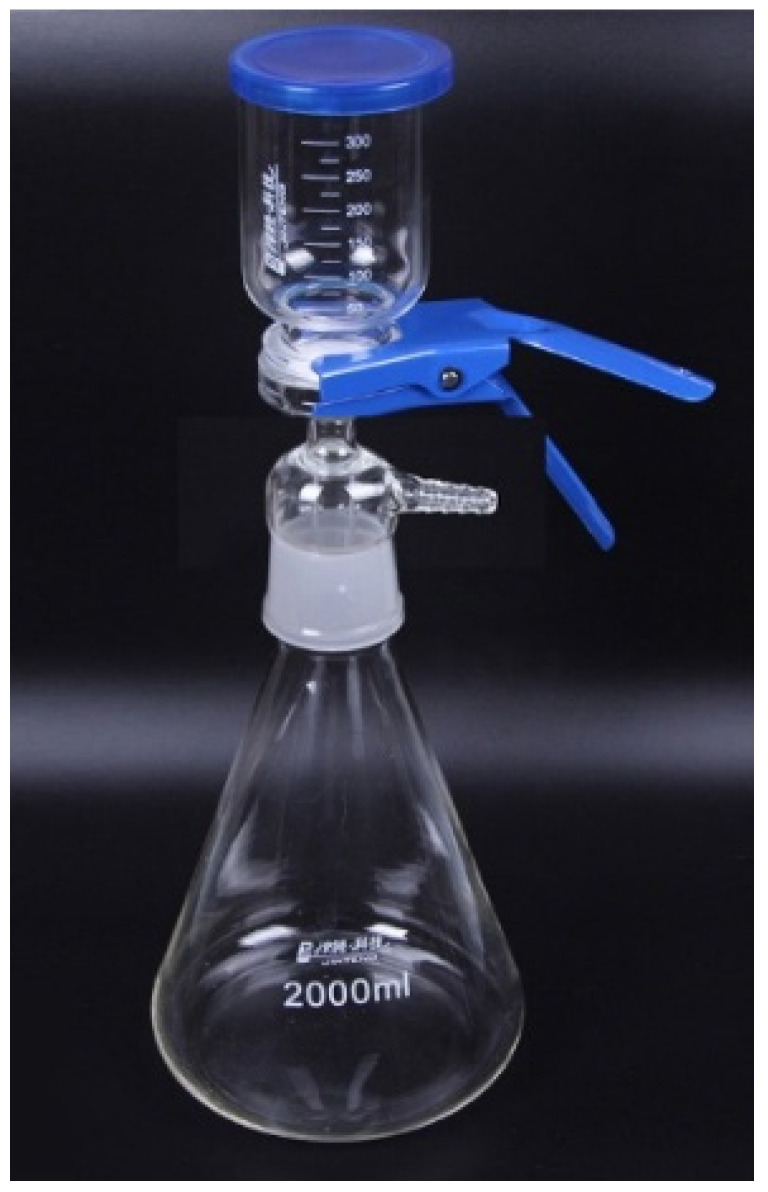
Sand core suction filter device.

The oil–water separation equipment was a sand core suction filter device, as shown in Figure 2. The top was the filter cup, and the oil–water mixed solution was added from above. The middle was the sand core filter, and the prepared Janus fiber membrane was placed between the filter cup and the filter. The fixing clip was used to fix the separation. Collect the separated liquid after separation. The presence of emulsion particles in the emulsion system was observed before and after separation with a polarizing microscope. When separating the mixture containing light oil, the hydrophobic side of the Janus cellulose acetate membrane was facing up to separate the water phase. When separating the mixture containing heavy oil, the hydrophilic side of the Janus cellulose acetate membrane was turned up to separate the oil. After each separation, the Janus cellulose acetate membrane was rinsed with deionized water 5 times to wash away the residue.

The membrane flux was measured by calculating the volume J (L/(m^2^·h)) of oil passing through the membrane per unit area per unit time. The volume J can be calculated according to the following equation (Equation (1))

(1) $$J=\frac{Q}{A•\Delta t}$$

where *J* denotes the permeate flux (L/m^2^ h), *Q* denotes the permeate volume (*L*). A denotes the effective permeate area of the fiber membrane (m^2^), and Δ*t* is the permeation time (h).

### 2.6. Interception Rate Test

The preparation methods of emulsion and oil–water mixture [37] are as follows: Take 2 mL of methylene blue aqueous solution and 0.5 g of Span 80 emulsifier and add 114 mL of toluene, and mechanically stir at 1000 rpm/min for 3 h to obtain a water-in-toluene emulsion. Water and 1,2-dibromoethane mixture: dye 1,2-dibromoethane with Disperse Red FB and dye deionized water with methylene blue. Mix the two as an oil–water mixture. Water/n-hexane mixed liquid: mix the methylene blue aqueous solution with n-hexane to prepare a mixed liquid of water and n-hexane.

(a) The separation rejection rate of the fiber membrane for the mixture of deionized water and 1,2-dibromoethane is calculated by the weighing method [38]. The rejection rate (*R*_1_) of the fiber membrane can be calculated according to the following equation (Equation (2)).

(2) $$R_{1}=\frac{m_{1}}{m_{0}}\times 100\%$$

where *m*_0_ is the water mass before separation (g); *m*_1_ is the water mass after separation (g).

(b) In order to calculate the separation rejection rate of the fiber membrane to the water-in-toluene emulsion, the UV-visible spectrophotometer was used to test the methylene blue dye concentration contained in the oil phase before and after the separation. The rejection rate (*R*_2_) was used to evaluate the oil–water separation performance, and *R*_2_ can be calculated according to the Equation (3).

(3) $$R_{2}=\frac{C_{m}-C_{n}}{C_{m}}\times 100\%$$

where *C_m_* denotes the concentration of methylene blue in the stock solution (g/L), *C_n_* denotes the concentration of methylene blue in the oil phase (toluene) (g/L).

## 3. Results and Discussion

### 3.1. Analysis on the Influence of Spinning Parameters of Centrifugal Spinning on the Morphology of CA Fiber

In the process of centrifugal spinning, the concentration of the spinning solution has a significant effect on the fiber morphology. In order to explore the influence of CA spinning solution concentration on fiber morphology, a CA spinning solution with a spinning solution concentration of 16–20 wt% was selected. The spinning speed was 10,000 r/min and the pore diameter was constant at 0.3 mm.

**Figure 3:**
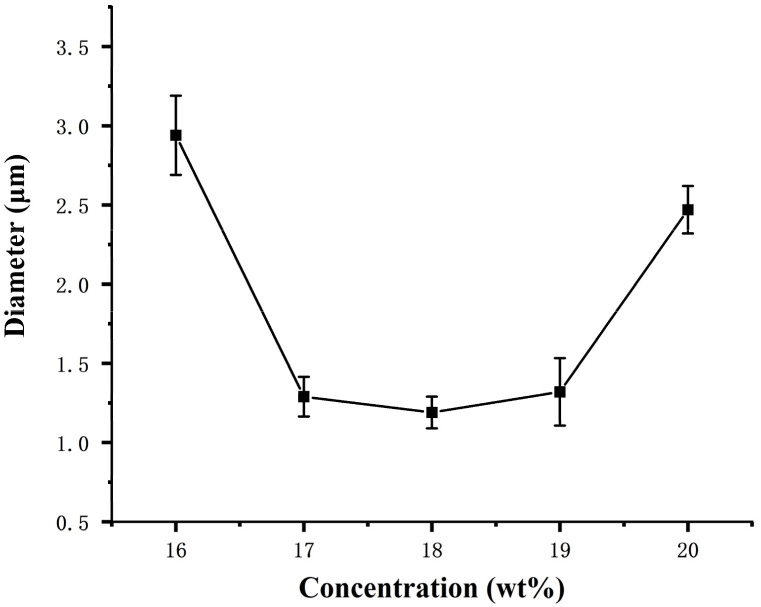
Variation curve of CA fiber diameters with spinning concentration solution.

It can be seen from the Figure 3 that as the concentration of the CA spinning solution increases, the fiber diameter first becomes smaller and then becomes larger. The average diameter of CA fibers with concentrations of 16, 17, 18, 19, and 20 wt% are 2.94, 1.29, 1.19, 1.32, and 2.47 μm, respectively. It can be seen that when the concentration of the spinning solution is below 18 wt%, as the concentration of the spinning solution increases, the fiber diameter becomes smaller, and the standard deviation of the fiber diameter is smaller than other concentrations. When the concentration of the spinning solution is above 18 wt%, as the concentration of the spinning solution increases, the fiber diameter becomes larger, but the standard deviation of the fiber diameter is larger than that of other concentrations. This is because when the concentration of the spinning solution is less than 17 wt%, the concentration of the spinning solution is too low, and the fiber is not easy to become filaments. During the spinning process, it is received in the form of a lump on the collecting rod, and the solvent accounts for a relatively large amount and cannot be volatilized in time. Completely, the prepared fibers are entangled and merged during the collection process, and a beaded and strip-like structure appears, which increases the average diameter of the fibers. When the concentration of the spinning solution is 18 wt%, the prepared CA fibers are uniform in thickness and fiber diameter. The distribution is concentrated, but when the concentration of the spinning solution is greater than 18 wt%. As the concentration of the spinning solution increases, the viscosity gradually increases. This results in a longer stress relaxation time for the solution and limiting the solvent’s evaporation, hindering the elongation and thinning of the jet. Finally, a fiber with a larger diameter forms.

**Figure 4:**
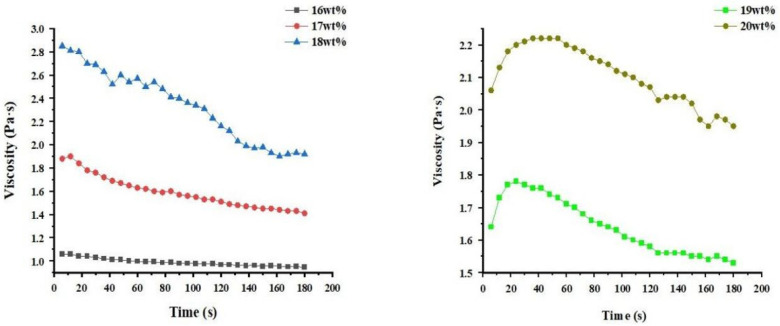
The variation curve of the viscosity of the spinning solution with the concentration.

From Figure 4, it can be seen that at a constant shear rate of 500 S^−1^, the concentration of the spinning solution gradually stabilizes over time. When the concentration of the solution increases from 16 wt% to 18 wt%, the viscosity of the solution as a whole becomes larger. Therefore, when the spinning solution concentration is 18 wt%, a smooth, non-blocking and uniform fineness centrifugal spun CA fiber can be obtained. However, when the concentration of the spinning solution exceeds 18 wt%, the viscosity of the spinning solution suddenly decreases. Moreover, the concentration of the spinning solution at a constant shear rate is no longer stable with time. This unstable variation in viscosity makes the diameter of the spun fibers uneven.

**Figure 5:**
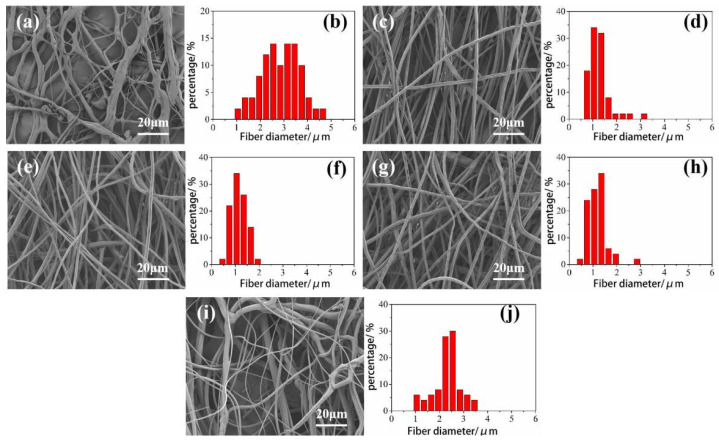
SEM images of fibers spun at different polymer concentrations and their corresponding diameter distribution. (**a**,**b**): 16 wt%; (**c**,**d**): 17 wt%; (**e**,**f**): 18 wt%; (**g**,**h**): 19 wt%; (**i**,**j**): 20 wt%.

In centrifugal spinning, huge centrifugal force and shear force are in the process of high-speed rotation, and different rotation speeds produce different centrifugal forces. The spinning solution overcomes surface tension under the action of high-speed centrifugation to form a jet. In order to explore the influence of the spinning speed on the fiber morphology, the spinning speed is selected as 6000, 8000, 10,000 r/min, the pore diameter is 0.3 mm, and the spinning solution concentration is 18 wt%. After spinning, the morphology and diameter distribution of the CA fiber membrane obtained, as shown in Figure 5.

**Figure 6:**
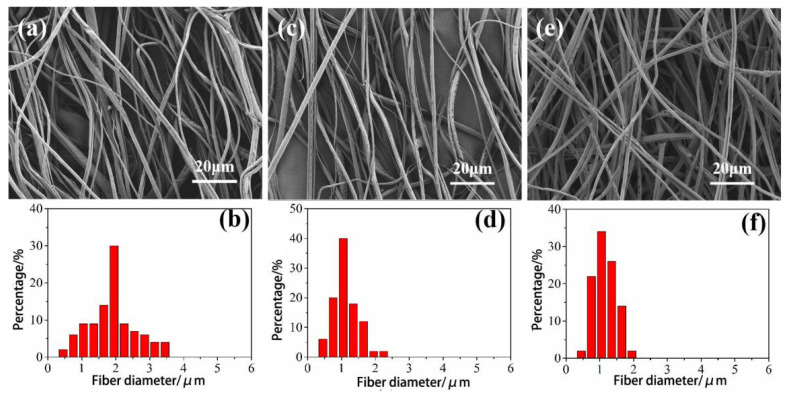
SEM images and fiber diameter distribution diagrams of CA fibers spun at different speeds. (**a**,**b**): 6000 r/min; (**c**,**d**): 8000 r/min; (**e**,**f**): 10,000 r/min.

It can be seen from Figure 6 that as the spinning speed increases, the fiber diameter becomes smaller. The average diameter of the CA fiber spun at the spinning speed of 6000, 8000, and 10,000 r/min is 1.95, 1.21, and 1.19 μm. This may be because as the rotation speed increases, the centrifugal force is larger, and the stretching degree of the jet increases such that the fibers are stretched thinner. At the same time, the fibers are not easily adhered to each other, which makes the spun fibers thinner. Therefore, when the spinning speed is 10,000 r/min, the CA fiber obtained by centrifugal spinning has uniform thickness, concentrated diameter distribution and good appearance.

**Figure 7:**
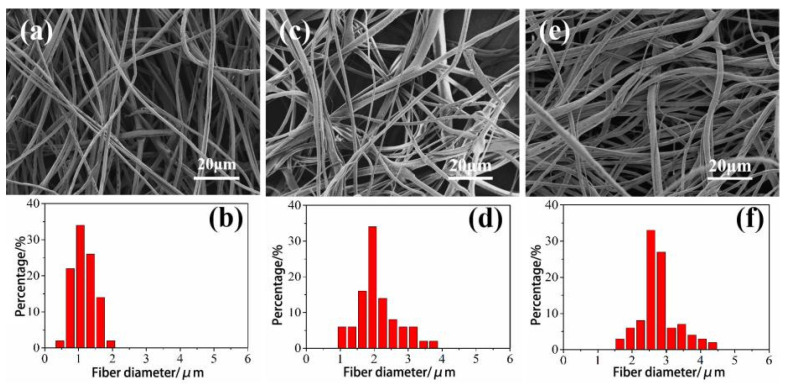
SEM images and fiber diameter distribution diagrams of CA fibers spun with different spinning head apertures. (**a**,**b**): 0.3 mm; (**c**,**d**): 0.4 mm; (**e**,**f**): 0.5 mm.

The aperture of the spinning head controls the flow of the spinning fluid jet, which has a greater impact on the morphology of the fiber. Figure 7 shows the SEM images and fiber diameter distribution diagrams of CA fibers spun with different spinning head apertures. Spinning heads with pore diameters of 0.3, 0.4, and 0.5 mm were selected in the experiment, and spinning was performed under the conditions of spinning speed of 10,000 r/min and spinning solution concentration of 18 wt%. The average diameter of the CA fiber spun with the diameter of the spinning head of 0.3, 0.4, and 0.5 mm are 1.19, 1.98, and 2.65 μm, respectively. This is due to the increase of the spinning fluid jet with the increase of the pore diameter, resulting in the inability of organic solvents. The volatilization is completed quickly, and the fibers cannot be stretched enough. The fibers are easier to adhere to each other, which makes the fiber diameter larger. Therefore, when the diameter of the spinning head is 0.3 mm, the CA fiber obtained by centrifugal spinning is thinner and has better linearity.

These are optimal process conditions for the preparation of CA fiber membrane by centrifugal spinning when the spinning solution concentration is 18 wt%, the spinning speed is 10,000 r/min, and the spinning head diameter is 0.3 mm. This work selects the process parameters to prepare Janus–CA fiber membranes for subsequent experiments and tests.

### 3.2. The Effect of Plasma Grafting D4 on Fiber Morphology and Wetting Properties

**Figure 8:**
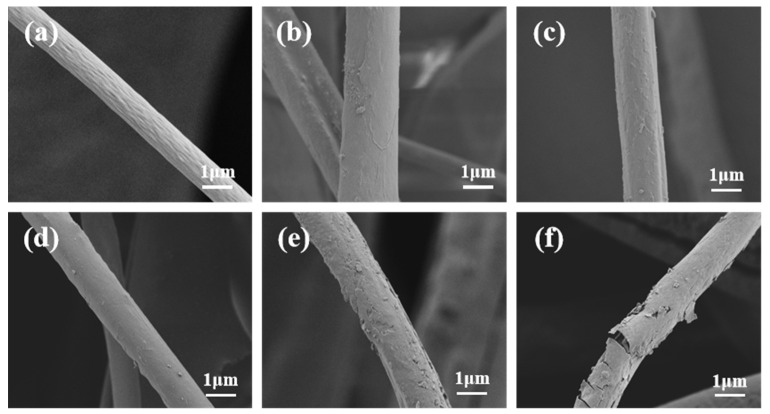
SEM images of centrifugal spinning CA fiber before and after plasma treatment: (**a**) control CA; (**b**) 3 min, 40 W; (**c**) 5 min, 60 W; (**d**) 8 min, 80 W; (**e**) 10 min, 80 W; (**f**) 10 min, 100 W.

In order to explore the influence of the process parameters of plasma grafting D4 monomer on the surface wettability of the centrifugal-spun CA fiber membrane, the CA fiber membrane was treated with a power of 40–100 W for 3–10 min. Figure 8 is the SEM image of centrifugal spun CA fiber before and after plasma grafting D4 treatment. As shown in Figure 8a, on the surface of the control CA fiber, obvious microwrinkles can be observed. Comparing Figure 8b,c, it can be observed that as time and power increase, the hydrophobic polymer film grafted on the surface of the fiber will gradually be completely covered. When the power is too large, the hydrophobic polymer film grafted on the surface of the fiber will gradually be completely covered. When the power is too high, the polymer film will be destroyed. The higher the power, the more serious the damage will be. When the processing condition is 100 W, it is obvious that the fiber surface is covered. The covering film peeled off (Figure 5e,f). From the perspective of fiber surface grafting, the treatment condition is 80 W. At 8 min, more monomers are grafted on the CA fiber surface, and the film coating is relatively complete (Figure 8d).

**Figure 9:**
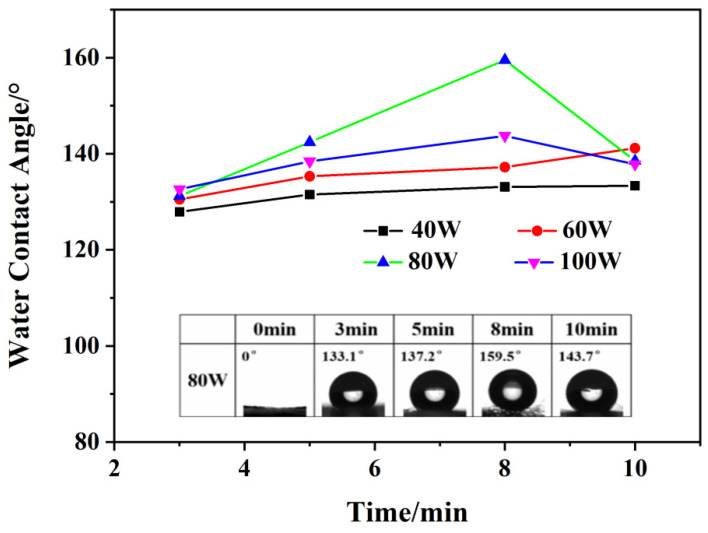
Water contact angle on the surface of CA fiber membrane after different plasma treatment (inset: water contact angle of the hydrophobic surface of CA fiber treated with different grafting time (80 W) and surface of untreated CA fiber).

The surface water contact angle of the CA fiber membrane after different plasma treatment is shown in Figure 9. It can be seen from Figure 9 that the surface of the control centrifugal spun CA fiber membrane still has good hydrophilicity. The maximum water contact angle of the CA fiber membrane obtained when the treatment power is 80 W and the time is 8 min is 159.5°, which achieves the superhydrophobic effect. When the treatment time is constant and the treatment power is less than 80 W, the contact angle of the CA hydrophobic surface increases with the increase of the treatment time. When the treatment power is greater than or equal to 80 W, the contact angle of the CA hydrophobic surface first increases and then decreases with the increase of the treatment time. When the treatment power is constant and the treatment time is 3 min, the contact angle of the CA hydrophobic surface gradually increases with the increase of the treatment power. However, when the treatment time is greater than 5 min, with the increase of the treatment power, the contact angle of the Janus–CA hydrophobic surface first increases and then decreases. This is because when the time and power are too high, the polymer film on the fiber surface is etched by high-energy electrons, which causes the polymer film deposited on the fiber surface to peel off, reducing the contact angle of the fiber film surface.

**Figure 10:**
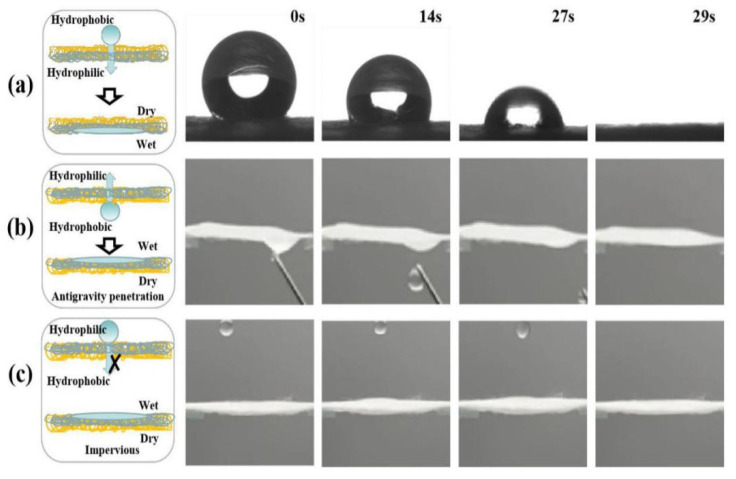
Centrifugal spinning Janus–CA fiber membrane the unidirectional water transfer diagram: (**a**) water droplets penetrate from the hydrophobic surface to the hydrophilic surface; (**b**) reverse gravity penetration; (**c**) water droplets spread on the hydrophilic surface.

When the plasma conditions are 5 min and 40 W, the Janus–CA prepared has the effect of directional humidity, as shown in Figure 10. It can be seen from Figure 10a that the water droplets begin to penetrate the hydrophilic surface in a round manner after contacting the hydrophobic surface, and the water droplets completely penetrate into the hydrophilic surface after 29 s. This is because under this treatment condition, the CA fiber surface has not yet formed a complete hydrophobic film. The untreated surface of the fiber membrane has good hydrophilicity, and the water droplets from the hydrophobic surface penetrate to the hydrophilic surface under the action of the Laplacian pull of the hydrophilic surface [39]. Water droplets can penetrate from the hydrophobic surface of the Janus–CA fiber membrane to the hydrophilic surface under anti-gravity conditions, as shown in Figure 10b. As can be seen from Figure 10c, the water droplets from the hydrophilic surface. When dripping, it spreads on the surface of the fiber but cannot drip through the hydrophobic surface, which shows that the fiber membrane has the characteristic of directional moisture conduction.

### 3.3. Surface Chemical Structure Analysis

**Figure 11:**
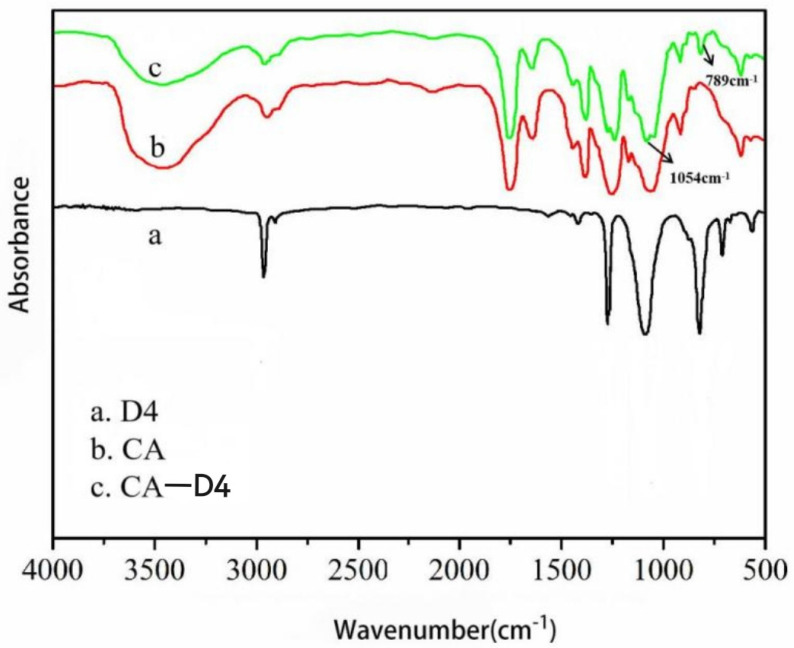
Infrared spectrum of CA fiber after grafting D4.

In order to explore the chemical bonds on the surface of CA fiber before and after grafting D4, the infrared spectra of D4, CA and CA after grafting D4 were tested, as shown in Figure 11. In the infrared spectrum (curve a) of D4 in Figure 11, the C-Si stretching vibration peaks, Si-O-Si antisymmetric stretching vibration peaks and C-Si bond deformation vibration peak was observed at the wave numbers of 1260, 1058, and 810 cm^−1^, respectively. In the spectrum of CA fiber (curve b), there is a broad absorption peak at 3464 cm^−1^, which belongs to the stretching vibration peak of O-H. The sharper absorption peak at 1753 cm^−1^ is the ester carbonyl C=O stretching vibration. In addition, the symmetric vibration peak of -CH_3_ is at 1378 cm^−1^, and the vibration absorption peak of acetyl ester bond is at 1244 cm^−1^ [40,41]. Comparing the curve b of CA fiber with the curve c of CA after 8 min and 80 W grafting D4, it can be seen that the CA after D4 graft copolymerization has a new Si-CH_3_ bond deformation vibration peak at 789 cm^−1^ and 1054 cm^−1^. With the Si-O-Si stretching vibration peak, the intensity of the hydroxyl group decreased in the infrared spectrum of CA after grafting, indicating that the D4 monomer reacted with the groups on the surface of the CA fiber and successfully grafted to the surface of the CA fiber [42].

**Figure 12:**
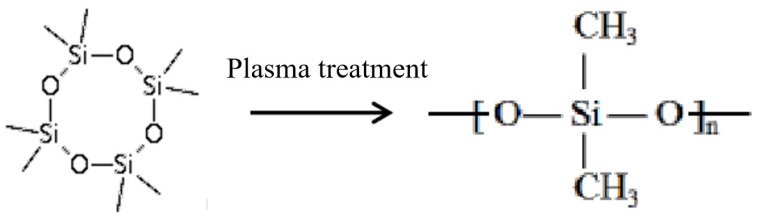
Schematic diagram of plasma graft polymerization D4.

New chemical bonds appear on the surface of CA fiber modified by plasma grafting. This is due to the polymerization reaction of the silicon-oxygen ring in the D4 molecular chain under the conditions of plasma glow discharge. Under the high-energy bombardment of plasma glow discharge, D4 molecules break off the silicon-oxygen ring and generate active groups. With the increase of plasma treatment time, these active groups polymerize to gradually form macromolecular polymers and gather in surface of CA fiber. The reaction on D4 is shown in Figure 12.

**Figure 13:**
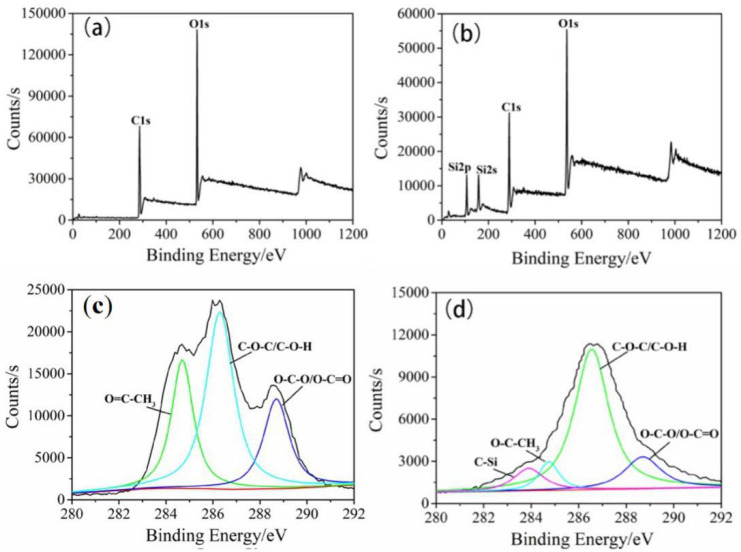
XPS spectrum: (**a**) Full spectrum of CA, (**b**) Full spectrum of Janus–CA hydrophobic surface, (**c**) C1s peak split: CA, (**d**) C1s peak split: Janus–CA hydrophobic surface.

The XPS spectra of the hydrophobic surface of CA and Janus–CA are shown in Figure 13. Figure 13a,b is the XPS full spectrum of the Janus–CA hydrophobic surface obtained by the treatment of CA and 80 W for 8 min. In Figure 13a,b, it can be seen that the CA fiber only has two peaks of C and O. After D4 plasma treatment, the hydrophobic surface of Janus–CA after grafting D4 contains three elements: C, O and Si. Figure 13c shows the XPS peaks of CA fiber C1s. The peaks at 284.7, 286.3, 288.7 eV are composed of acetyl CH3 part, C−O−C and C−O−H part. The C atoms in the O−C−O and O−C=O parts are generated. In Figure 13d, a new peak appears in the C1s peak pattern of the Janus–CA hydrophobic surface after plasma treatment. That is, the C−Si bond is at 283.9 eV, indicating that Si element is introduced to the surface of CA fiber after plasma treatment. 

### 3.4. Janus–CA Fiber Surface EDS Analysis

**Figure 14:**
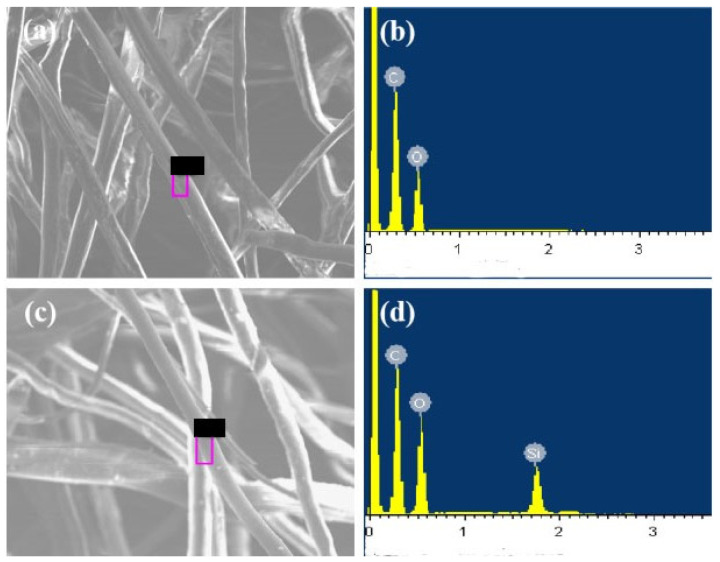
EDS analysis: (**a**,**b**) CA fiber; (**c**,**d**) Janus–CA fiber.

The surface elements of CA and Janus–CA fiber are investigated by using EDS. As shown in Figure 14, the surface of CA fiber contains two elements: C and O. After D4 plasma grafting for 8 min, the element Si appears on the surface of Janus–CA fiber after 80 W polymerization. Table 1 shows the percentage of elements in the hydrophobic surface of CA and Janus–CA fiber. The content of C and O elements on the surface of CA are 56.66% and 43.34%, respectively. The percentage of C and O elements on the surface of Janus–CA fiber are 50.27% and 38.13%. The percentage is 11.60%, which further illustrates that D4 monomer polymerizes on the surface of CA fiber after plasma treatment.

### 3.5. Janus–CA Fiber Membrane Pore Size Test

**Figure 15:**
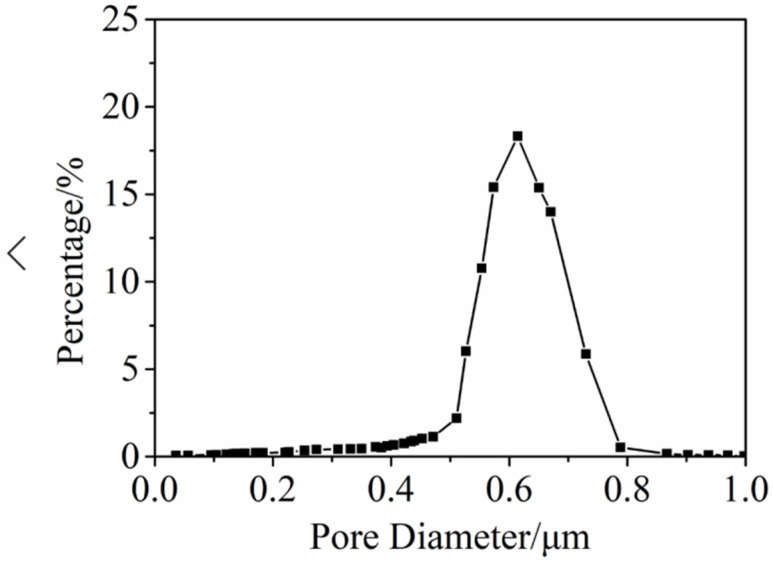
Janus–CA fiber membrane pore size distribution curve.

The pore size of the membrane is an important parameter to evaluate the performance of the membrane, and the development of the membrane pore mainly depends on the choice of suitable polymer. We used a sample with a concentration of 18 wt% that performs better in all aspects for testing to observe its pore size distribution. The pore size and distribution of superhydrophobic/hydrophilic Janus–CA fiber membrane were tested. As shown in Figure 15, the pore size distribution of Janus–CA fiber membrane is mainly concentrated in the range of 0.5~0.8 μm, with an average pore size of 0.63 μm. It reaches the pore size range of the microfiltration membrane.

### 3.6. Janus–CA Fiber Membrane Oil–Water Separation Performance Test

The superhydrophobic/hydrophilic Janus–CA fiber membrane and the unidirectional water transfer Janus–CA fiber membrane are used to separate the oil phase in the water-in-toluene emulsion, water and 1,2-dibromoethane mixture, and the water in the water and n-hexane mixture, respectively.

**Figure 16:**
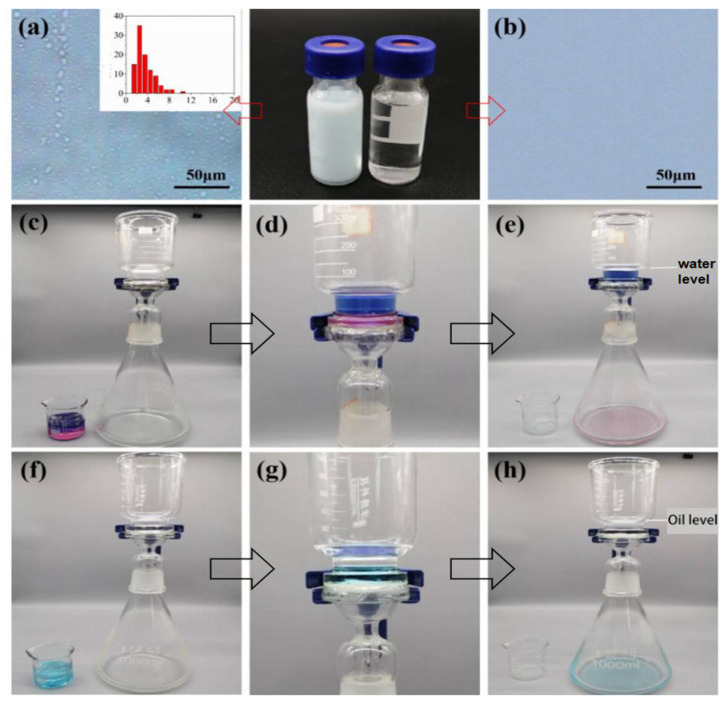
Janus–CA oil–water separation diagram. (**a**,**b**) Separation of the water-in-toluene emulsion, (**c**–**e**) Separation of the water and 1,2-dibromoethane mixture, (**f**–**h**) Separation of the water and n-hexane mixture.

Figure 16a,b is the polarizing microscope pictures of the water-in-toluene emulsion before and after the separation. Figure 16a shows the particle diameter distribution of the water emulsion it can be seen that the particle diameter of the water-in-toluene emulsion before separation is in the range of 1~11 μm. The emulsion particles are evenly distributed in the water-in-toluene emulsion before separation, but there are no emulsion particles in the separated liquid (Figure 16b). The separated liquid is clear and transparent. Figure 16c–e are diagrams that superhydrophobic/hydrophilic Janus–CA fiber membrane separates water and 1,2-dibromoethane mixture. Figure 16f–h shows diagrams of unidirectional water transfer Janus–CA fiber membrane separates n-hexane and water mixture. It can be observed that after the separation of the fiber membrane, the blue water phase and the two oil phase substances are separated. The unidirectional water transfer Janus–CA fiber membrane has good hydrostatic pressure resistance. The unidirectional water transfer Janus–CA’s hydrophilic surface is injected with dyed deionized water. When the water volume reaches 35 mL, the fiber membrane can still withstand hydrostatic pressure.

### 3.7. Membrane Flux Test

**Figure 17:**
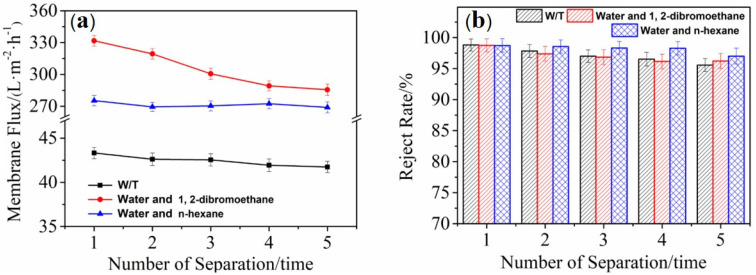
(**a**) Variation curve of the membrane flux of Janus–CA fiber membranes with the number of separations. (**b**) The interception rate of Janus–CA fiber membranes with the number of separations.

The separation ability and recycling performance of the sample for oil–water mixtures were investigated. The flux of Janus–CA fiber membrane separation was tested for 5 times. The separated fiber membrane was washed with ethanol and distilled water and dried at 60 °C for recycling. Figure 17a shows the separation of water-in-toluene emulsion, water and 1,2-dibromoethane mixed liquid with superhydrophobic/hydrophilic Janus–CA fiber membrane, and the membrane flow of the unidirectional water transfer Janus–CA separating water and n-hexane mixture. The membrane fluxes of the Janus–CA fiber membrane for the first separation of the three oil–water mixtures are 43.32 ± 0.65, 331.72 ± 5.11, and 275.27 ± 4.94 L/(m^2^·h), respectively. The membrane fluxes of the Janus–CA fiber membrane for the separation of three oil–water mixtures after five separations are 41.74 ± 0.63, 285.51 ± 5.32, and 268.94 ± 5.21 L/(m^2^·h), respectively. The flux of superhydrophobic/hydrophilic Janus–CA fiber membrane used to separate the oil phase decreases with the increase of the number of separations, and finally stabilizes. While the flux of the unidirectional water transfer Janus–CA fiber membrane for separating the water phase remains good stability. This is because the oil phase will contaminate the surface of the hydrophilic side of the superhydrophobic/hydrophilic Janus–CA fiber membrane and leave a small amount of oil stains in the fiber pores, which affects the membrane flux. However, due to the good performance of Janus–CA fiber membrane, the membrane flux can gradually stabilize with little change. In addition, it is difficult to separate emulsions with simpler mixtures, and demulsification will increase the burden of oil–water separation; thus, the membrane flux is the lowest. The unidirectional water transfer Janus–CA maintains a stable flux due to its good transmission of the water phase.

### 3.8. Rejection Rate Test

The rejection rate of Janus–CA fiber membrane for oil–water separation is shown in Figure 17b. The superhydrophobic/hydrophilic Janus–CA fiber membrane is the first interception of the water phase in the water-in-toluene emulsion, water and 1,2-dibromoethane mixture. The rates are respectively 98.81% ± 0.97% and 98.76% ± 1.05% (because 1,2-dibromoethane is slightly soluble in 250 times of water, the slightly soluble amount is included in the error value during measurement). The first rejection rate of Janus–CA to the oil phase in the mixture of water and n-hexane is 98.73% ± 1.09%. After five separation cycles, the rejection rates of Janus–CA fiber membrane for water and n-hexane mixture, water and 1,2-dibromoethane mixture, and water-in-toluene emulsion are 97.12% ± 1.29%, 96.23% ± 1.21% and 95.57% ± 1.06%, respectively. The directed and humidified Janus–CA which separates the water phase has good recyclability, and the average rejection rate drops by 1.61% after five separations.

## 4. Conclusions

In this work, Janus–CA was successfully prepared by hydrophobic modification of the CA membrane surface obtained by centrifugal spinning. When CA membrane is treated by plasma for 8 min and 80 W, the water contact angle of the CA fiber membrane surface reaches 159.5°, and the superhydrophobic/hydrophilic Janus–CA fiber membrane can be prepared. When CA membrane is treated by plasma gas phase grafting by polymerizing octamethylcyclotetrasiloxane (D4) for 5 min and 40 W, the unidirectional water transfer Janus–CA fiber membrane can be prepared. Superhydrophobic/hydrophilic Janus–CA was used to separate water/toluene emulsion, water and 1,2-dibromoethane mixture. Unidirectional water transfer Janus–CA was used to separate n-hexane and water mixture. The separation for the first time interception rate of water and n-hexane mixture, water and 1,2-dibromoethane mixture, and water-in-toluene emulsion were 98.81% ± 0.97%, 98.76% ± 1.05% and 98.73% ± 1.09%, respectively. After five times of separation, the interception rate was 95.57% ± 1.06%, 96.23% ± 1.21% and 97.02% ± 1.29%, respectively. It has well repeated separability. At the same time, the Janus–CA membrane also provides a simple and efficient method for preparing oil–water separation membranes. All these results confirm the effectiveness of Janus–CA membranes for oil–water separations.

## Figures and Tables

**Table 1:** CA and Janus–CA fiber surface element weight percentage.

Element Content Ratio	C/%	O/%	Si/%
CA	56.66	43.34	/
Janus–CA hydrophobic surface	50.27	38.13	11.60

## Data Availability

Not applicable.
